# Transition Metal Nitrides for Electrocatalytic Application: Progress and Rational Design

**DOI:** 10.3390/nano12152660

**Published:** 2022-08-03

**Authors:** Zihan Meng, Shuhong Zheng, Ren Luo, Haibo Tang, Rui Wang, Ruiming Zhang, Tian Tian, Haolin Tang

**Affiliations:** 1 Foshan Xianhu Laboratory of the Advanced Energy Science and Technology Guangdong Laboratory, Xianhu Hydrogen Valley, Foshan 528200, China; mengzihan@xhlab.cn (Z.M.); tanghaibo@xhlab.cn (H.T.); wangrui@xhlab.cn (R.W.); zhangruiming@gdh2ri.org.cn (R.Z.); 2 State Key Laboratory of Advanced Technology for Materials Synthesis and Processing, Wuhan University of Technology, Wuhan 430070, China; 246027@whut.edu.cn (S.Z.); 257229@whut.edu.cn (R.L.); 3 Guangdong Hydrogen Energy Institute of WHUT, Xianhu Hydrogen Valley, Foshan 528200, China

**Keywords:** transition metal nitrides, synthesis methods, electrocatalytic application, oxygen reduction reaction, oxygen evolution reaction

## Abstract

The energy crisis and environmental issues are becoming more severe due to the long-term consumption of fossil fuels. Therefore, novel energy-conversion devices with high energy density and environmental friendliness are expected to provide reliable alternatives to traditional fossil-based energy systems. However, because of the inevitable use of costly precious metals as the electrode catalysts for such devices, their popularization is seriously hindered. Transition metal nitrides (TMNs) exhibit similar surface and adsorption properties to noble metals because the atomic distance between metal atoms increases and the d-band center of metal atoms downshifts after nitrogen atoms enter the metal lattice. TMNs have become one of the best electrode materials to replace noble metal-based electrocatalysts in next-generation energy-storage and energy-conversion devices. In this review, the recent developments in the electrocatalytic application of TMNs are covered. First, we discuss the structure and activity origin of TMNs and introduce the common synthesis methods for the preparation of TMNs. Subsequently, we illustrate the applications of mono-metallic TMNs and multi-metallic TMNs in oxygen-reduction reaction, oxygen-evolution reaction, and bifunctional oxygen reduction and evolution reactions. Finally, we summarize the challenges of TMNs encountered at the present stage, and expect their future development.

## 1. Introduction

The rapid depletion of energy sources and the decline of fossil fuels, together with the consequential environmental crisis have driven urgent demands to develop green and renewable energy systems [1,2,3,4,5]. The next generation of energy-storage and energy-conversion devices, such as fuel cells and metal–air batteries, are emerging on account of their high energy density, environmental friendliness, and good security [6,7,8,9]. The operational performance of such devices is largely determined by the catalytic electrode; the choice of electrocatalyst is vital for practical application [10,11,12]. Nevertheless, the sluggish kinetics of their electrochemical reaction is noticeable. This is especially true of oxygen-reduction reaction (ORR) and oxygen-evolution reaction (OER), which has multiple electron transfer steps and requires precious metals to reduce overpotential and guarantee operational efficiencies. These precious metals include Pt/Ir-based catalysts, which have prohibitive costs and are scarce; this has enormously hindered large-scale commercialization [13,14]. Considering this, numerous efforts have been made to explore electrocatalysts based on earth-abundant elements for getting rid of the dependence on precious metals [15,16,17,18].

Transition metal nitrides (TMNs), formed by the insertion of electronegative nitrogen atoms into the interstitial sites of the parent species, are characterized by covalent compounds, ionic crystals, and transition metal features [19,20]. As a kind of non-noble metal material, TMNs exhibit similar surface and adsorption properties to the VIII group of precious metals (such as Pt and Pd) because the atomic distance between metal atoms increases and the d-band center downshifts after the incorporation of nitrogen atoms [21,22]. Combined with attractive electrical conductivity, robust chemical stability, and remarkable mechanical robustness, TMNs have great potential as high-efficiency catalysts in various areas such as electrocatalysis, hydrogenation/desulfurization of fuel oil, synthesis/decomposition of ammonia, and other fields [23,24,25,26]. Moreover, their impressive chemical inertness and high corrosion resistance allow TMNs to be applied in a wide array of pH conditions for long periods, expanding the electrocatalytic application in various mediums [27,28].

In recent years, researchers have found that downsizing materials to the nanoscale can usually give them unusual mechanical, electrical, and optical properties [29,30]. Hence, the study of nanostructured electrocatalyst materials with tiny nanoparticles has aroused great interest. It is well-known that ideal electrode materials should possess excellent abilities for electronic conductivity and ionic conductivity. The rational design of TMNs’ nanocrystalline-based electrocatalysts can not only notably reduce the consumption of precious metals, but also improve the ionic conductivity of materials by shortening the diffusion length of ions in the electrocatalytic process. Therefore, the design of favorable nanostructures for electrode materials can significantly improve electrocatalytic performance [31,32,33]. The unique nanostructure will provide new features of the electrocatalytic surface, which can significantly enhance the activity of the electrocatalyst, and lead to a large specific surface area and more adsorption and reaction sites [34]. As a result, the rational design of TMN-based materials is an important research field for electrode catalysts of energy storage and conversion devices.

In this review, we aim to collate and highlight the most used preparation methods and structural characteristics of TMNs, with outstanding performance and promising applications in the next-generation energy storage and conversion devices. In addition, the prospects of TMN applications in electrochemical reactions such as ORR, OER, and bifunctional oxygen reactions (a catalyst display the electrocatalytic abilities for both ORR and OER) are discussed in detail. At the end of this review, the major opportunities and challenges for further research directions on TMN-based materials are proposed.

## 2. Synthetic Methods and Structural Properties of TMNs

**Figure 1:**
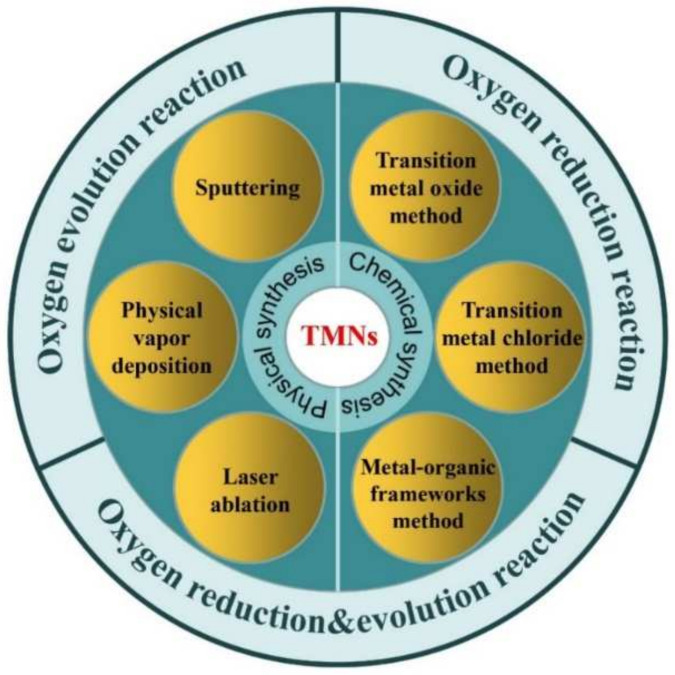
Simple diagram of the synthetic methods and applications of TMNs.

Due to the outstanding electrocatalytic performance, TMNs have undoubtedly garnered much attention and interest for researchers and organizations. However, the studies and discussions on the preparation strategies are relatively few at present, which impedes the rapid development of TMNs. The synthetic synthesis methods of TMNs can be mainly divided into physical methods and chemical methods (Figure 1). The physical methods mainly involve laser ablation, sputtering, arc discharge, physical vapor deposition, etc., whereas the chemical methods generally involve direct nitriding of transition metals, nitriding of transition metals oxides, ammonolysis of transition metals chloride, solvothermal method, thermal decomposition of polymer precursors, and so on.

### 2.1. Physical Synthetic Methods

The most common synthetic method in physical mode is deposition, whereby the sample is mainly obtained in the form of a thin film. That is, different physical means are used to load the reactants onto the substrate. Physical vapor deposition (PVD) technology is a relatively popular deposition method at the present stage. PVD refers to the use of physical processes (such as thermal evaporation of material) to prompt the surface of source material to vaporize under vacuum conditions. Atoms, molecules, or partially ionized ions are then deposited to form a film with some special functions on the surface of the substrate through low pressure, thus realizing the control of atom transfer from source materials to coatings.

PVD technology is always combined with other physical strategies (such as sputtering, ion irradiation, etc.) to synthesize TMNs. Sputtering is the most commonly used physical synthesis method due to its high deposition speed, which requires the sputtering gas (argon) and the reaction gas (nitrogen) to prepare high-purity thin film samples with controllable stoichiometry and composition by PVD. For instance, Zhu et al. successfully deposited cubic, wurtzic, and explosive high-pressure phases of boron nitride (BN) films on the metal alloy substrates by tuned substrate radio frequency magnetron sputtering and PVD techniques [35]. The percentage of cubic boron nitride phase in the film was about 50% as calculated by Fourier transform infrared spectroscopy measurements. Compared with the cubic phase formed by the traditional low-energy ion bombardment, the prepared cubic boron nitride phase has low internal stress, which could largely solve poor adherence and delamination from the substrates. Recently, NiN thin films with a cauliflower shape and tetrahedral crystal lattices were developed by reactive sputtering in the N_2_ atmosphere [36]. Later, bimetallic carbon paper supported MoVN thin films by magnetron co-sputtering were reported [37], and a series of Mo_3_N_2_, Ag-Mo_3_N_2_, V-Mo_3_N_2_, and CuMo_3_N_2_ films were designed by the magnetron co-sputtering technique [38].

In addition to sputtering, PVD is also used in combination with ion irradiation, known as ion mixing and vapor deposition (IVD), which can easily change and design the physical and chemical properties of thin TMN films. For example, titanium aluminum nitride (Ti, Al)N films, by depositing Ti and Al metal vapor under simultaneous irradiation by nitrogen ions, were prepared [39]. Liu et al. also successfully prepared a novel quaternary (Ti, Al, Zr)N coating on Si_3_N_4_ ceramic substrates by using the PVD technology and multi-arc ion plating technique [40]. The (Ti, Al, Zr)N-coated Si_3_N_4_ cutting tools prepared at the gas pressure of 2.5 Pa had the most extended lifetime and the best mechanical performance. Although these physical methods can yield a definite structure to TMNs, the conditions of such physical synthetic methods are excessively harsh, and the preparation process is cumbersome. Such physical methods are not suitable for the general synthesis of TMNs.

### 2.2. Chemical Synthetic Methods

The chemical synthesis of TMNs generally employs temperature-programmed reactions and usually includes two steps. First, the precursors of TMNs are synthesized with different methods. Then the metal precursors undergo nitridation at NH_3_ or N_2_/H_2_ atmosphere under different temperatures to obtain diverse TMNs [41]. According to the different types of precursors, the chemical synthesis for TMNs can be divided into the transition metal oxide method, transition metal chloride method, metal-organic frameworks method, and other methods.

#### 2.2.1. Transition Metal Oxide Method

Transition metal oxides (TMOs), as the common compound form of transition metal, are commonly used as precursors to proceed with nitridation for TMN preparation [42]. Recently, hot ammonia reduction heat-treated at a moderate temperature of 300–800 °C was developed to obtain TMNs with different dimensions. The morphology of obtained TMNs is mainly inherited from TMOs, except those external mesopores derived from the volume shrink due to the nitrogen atom replacing the oxygen atom in the lattice. The high porosity of TMNs prompts the mass transfer in their downstream application.

**Figure 2:**
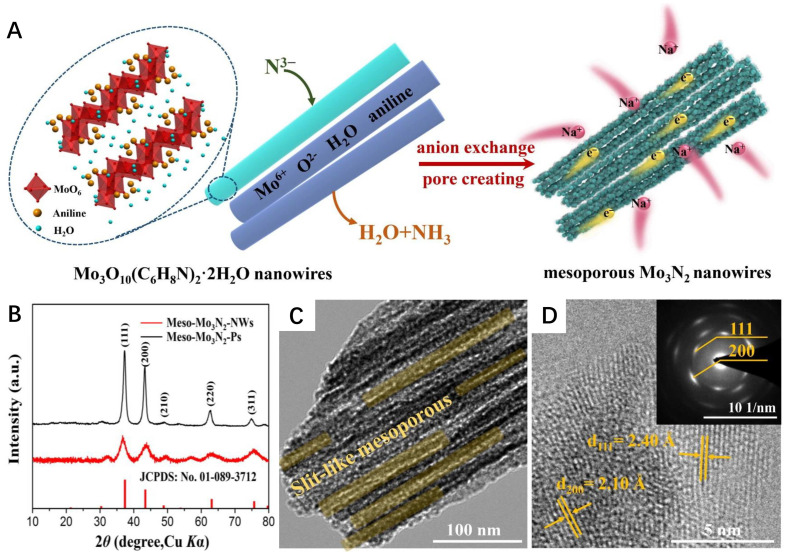
(**A**) Schematic of the formation of Meso-Mo_3_N_2_-NWs; (**B**) XRD patterns of the Meso-Mo_3_N_2_-Ps and Meso-Mo_3_N_2_-NWs; TEM (**C**) and HRTEM (**D**) images of Meso-Mo_3_N_2_-NWs. Reproduced with permission from [40]. Copyright © 2021 Elsevier.

For example, Peng et al. transformed a TiO_2_ nanotube array into TiN, and used the resulting porous TiN nanotube as cores to prepare an external porous double-layer MoOx (MoO*_x_*/TiN/MoO*_x_*) nanotube with high conductivity [43]. The porous structure facilitates electrolyte infiltration and maximizes the exposure of active sites. Simultaneously, the high conductivity of TiN gave the material a high specific capacitance. A temperature-programmed reaction was employed to convert MoO_3_ to Mo_2_N in the atmosphere of NH_3_ and N_2_/H_2_ [44]. The synthesized electrocatalyst shows an irregular surface with abundant nanopores and high specific surface areas, leading to an excellent electrocatalytic performance in hydrodenitrification, hydrodesulfurization, hydrolysis, and hydrogenation. As shown in Figure 2A,B, a similar approach transformed MoO_3_ nanowires into mesoporous Mo_3_N_2_ nanowires with cubic crystal nitrogen vacancy after annealing in the NH_3_ atmosphere at 800 °C [45]. The TEM images exhibit abundant slit-like mesopores, and the resulting Mo_3_N_2_ nanowires with a large specific surface area display excellent specific capacity and cycle durability for sodium-ion storage electrodes (Figure 2C,D). In another report, Yang et al. synthesized VN, TiN, NbN, and Ta_3_N_5_ by passing ammonia through a pipeline containing the oxides of V, Ti, Nb, and Ta under the protection of argon gas at 450–800 °C [46]. However, such preparation processes are often time-consuming. To optimize the synthesis process and reduce the reaction time, a novel rapid nitriding process was reported [47]. A series of ordered mesoporous TMNs were synthesized by using mesoporous TMOs. The ordered mesoporosity promotes the interaction between ammonia and precursors, which effectively reduces the nitriding time. Moreover, such rapid nitriding also inhibits the closure and collapse of mesoporous structures during the nitriding process. In addition, the researchers found that amorphous TMOs could also be used to prepare TMNs. For example, the processing of metastable MoO_2_ through hydrazine reduction of (NH_4_)_6_Mo_7_O_24_ solution was proposed [48]. The amorphous MoO_2_ was converted into face-centered cubic γ-Mo_2_N at ammonia flow under 400 °C, and transformed into hexagonal σ-Mo_2_N at a higher temperature of 600 °C.

The method to prepare TMNs by employing TMOs as precursors is relatively mature and widely used. However, the process of such a strategy invariably involves the high-temperature treatment, in which TMOs tend to undergo crystal transformation or transition between crystalline and amorphous structure, resulting in inadequate nitriding of the transition metal and even affecting the final structure of TMNs.

#### 2.2.2. Transition Metal Chloride Method

As the reaction temperature of transition metal chloride and ammonia is lower than that of TMOs, it is also widely used for the synthesis of TMNs. For example, the preparation of tantalum and tungsten nitrides was reported through a two-step process [49]. First, the tantalum and tungsten chloride were ammonolyzed in anhydrous chloroform at room temperature. Then, the prepared powder was heat-treated in an ammonia atmosphere to obtain tantalum nitride (TaN and Ta_2_N) and tungsten nitride (WN) at 600 °C. Furthermore, alkaline-earth chloride nanoparticles were developed by chloridizing scheelite with CCl_4_. These nanoparticles were then ammonolyzed to obtain MoN and WN [50]. In this manner, the transition metal nitriding could be carried out at a moderate temperature (500–550 °C), which is much lower than the temperature for the traditional ammoniation of TMOs by using pure NH_3_ or N_2_/H_2_ mixtures.

In addition, the transition metal chloride method could also be suitable for yielding transition metal-nitride nanocomposites. For example, a novel strategy was developed for the preparation of mesoporous Pd/Si_3_N_4_ composite nanomaterials derived from the Si_3_N_4_ complex with palladium chloride and silicon diimide gel [51]. This universal method could also be used for the preparation of other M/Si_3_N_4_ composites (M = Ni, Co, Zr, Ru) with a high specific surface area. Various metal nitrides (such as CrN, Fe_2_N, etc.) were obtained by using metal-urea chloride as precursors, and this study also facilitated the understanding of the feasibility toward metal-urea chloride-based, low-temperature synthesis [52]. Moreover, the transform reaction was based on the nucleation growth mechanism. The use of the metal-based complex as precursors to prepare TMNs was also considered. For example, Weil et al. offered a method to prepare TMN powders and coatings [53]: transition metal chlorides were first dissolved in solvents, such as acetonitrile, and the mixture was reacted with alkanolamine to form a viscous chelating solution as the precursor for generating TMNs through heat treatment.

Although the temperature in the transition metal chloride method is lower than that of the transition metal oxide method, the tedious reaction steps and abundant organic reagents make it impossible for large-scale manufacture of TMNs.

#### 2.2.3. Metal-Organic Frameworks Method

**Figure 3:**
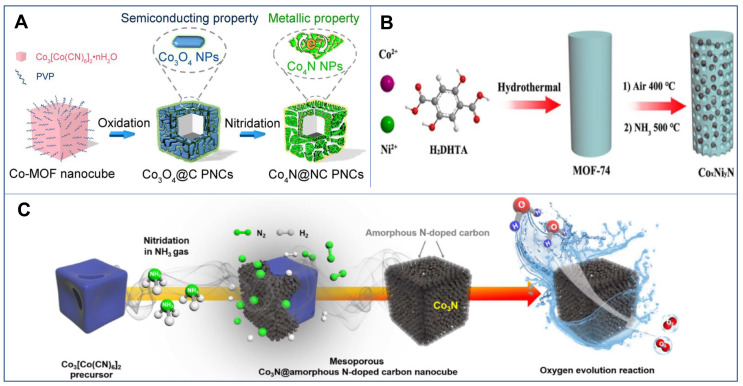
(**A**) Schematic of the synthesis route of Co_4_N@NC PNCs. (**B**) Schematic diagram of the formation of porous Co*_x_*Ni*_y_*N composites. (**C**) Schematic illustration of the synthesis process of mesoporous Co_3_N@amorphours N-doped carbon NCs. Reproduced with permission from [54,55,56]. Copyright © 2018 and 2019 American Chemical Society. Copyright © 2019 Tsinghua University Press.

In addition to transition metal oxides and chloride, metal-organic frameworks (MOFs) are also commonly used precursors for TMN preparation. MOFs are crystalline porous materials with periodic network structures formed by the self-assembly of transition metal ions or metal clusters with organic ligands through coordination bonds or other forces. Due to the unique porous structure of MOFs, TMNs derived from MOFs generally display a relatively high porosity [54,55,56]. As shown in Figure 3, Co*_x_*N*_y_* with various nanostructures were developed through different MOF-based precursors, and the resulting MOF-derived TMNs largely maintained porous features. In terms of electrocatalysis applications, the rich pore structure can increase the exposure of the active sites and boost the mass transfer rate.

In recent years, numerous reports developed TMNs derived from MOFs. For example, porous Co_3_FeN*_x_*/N-doped carbon nanotube arrays supported on carbon cloth were synthesized through simple nitriding of Fe-doped ZIF-67 [57]. The synthesized samples showed hierarchically porous structures, which endow superior electrocatalytic performance. Moreover, a novel method was reported for manganese nitride (MnN) by cracking manganese triazole in an oxygen-free environment [58]. Co@Co_4_N/MnO-NC was prepared by pyrolysis of the Mn-containing molecular sieve-imidazole framework [59]. A series of porous iron nitride (FeN) nanoparticles were obtained by using MOF-like cubic crystal Prussian blue as a precursor by a rapid nitridation process [60]. The FeN-based samples largely retained the nanostructure with a high specific surface area after the transformation process.

#### 2.2.4. Other Chemical Methods

In addition to the methods mentioned above, many other chemical synthetic strategies have been employed by researchers to synthesize TMNs. For example, Barker et al. dissolved transition metal into liquid zinc. The solution was then reacted with nitrogen to prepare binary and ternary nitrides [61]. Ultrathin 2D Mn_3_N_2_ was synthesized by salt-templating strategy, the Mn_3_N_2_ was yielded on the surface of salt due to the lattice matching mechanism [62]. Moreover, a simple and soft strategy was reported by employing urea as a nitrogen source to synthesize various transition metal nitride compounds (TiN, NbN, Mo_2_N, W_2_N, NbC*_x_*N_1*−x*_) with high yields [63]. In conclusion, abundant TMNs with diverse morphology have been prepared by scholars using other precursors and different nitriding methods. There are still many feasible approaches, which need to be discussed in the future.

## 3. Transition Metal Nitrides as Electrocatalysts

Transition metal nitrides are used as electrocatalysts due to their stable structure, high conductivity, and strong anti-poisoning ability [64]. On the one hand, as a kind of interstitial nanocrystal material, TMNs have the characteristics of covalent compounds, ionic crystals, and transition metals derived from the rearrangement of metal–metal bonds, the formation of metal and non-metal atoms, and the electron transfer between metal and non-metal atoms. On the other hand, the highly electronegative nitrogen species in the gap increase the metal atom spacing, leading to the shrinkage of the transition metal d-band as well as the increase of the Fermi level density of states. Therefore, the surface properties and adsorption characteristics of TMNs (Ti, Ni, Fe, Co, Mo, etc.) are similar to those of noble metals [65,66,67]. Thus, TMNs have high performance in the field of ORR, OER, bifunctional oxygen reactions, and so on.

### 3.1. Transition Metal Nitrides as ORR Electrocatalysts

At present, the oxygen reduction electrocatalytic process mainly relies on the Pt-based electrocatalyst. However, due to the shortage of Pt resources and the continuous increase in prices, the dependence of energy-conversion devices on precious metals has greatly restricted its industrialization process. TMNs are considered to be one of the most promising alternatives for noble metal ORR electrocatalysts due to their low price, abundant reserves, and comparable electrocatalytic performance [68,69].

#### 3.1.1. Mono-Metallic TMNs

Transition metal-nitrogen-based compounds were first discovered in 1964 as ORR electrocatalysts [70]. Jasinski experimentally found that cobalt, cooperating with nitrogen, showed encouraging activity for ORR. Since then, TMNs have been widely studied. Zheng et al. made a study about TMNs serving as the support of well-dispersed precious metals, and a 3D layered porous Pt/TiN electrocatalyst was synthesized by a simple approach [71]. The porous 3D support with higher corrosion resistance and mechanical stability played a key role in improving the conductivity and chemical durability of Pt species. As a result, Pt/TiN showed a more satisfactory electrochemical performance than Pt/C, which included a 40 mV negative shift in the half-wave potential (E_1/2_) and more robust durability. Similarly, a simple method was put forward to prepare mesoporous CrN by the ammonolysis of a bulk K_2_Cr_2_O_7_, and synthesized Pt/CrN electrocatalyst with mesoporous CrN as the support, addressing the corrosion issues for carbon support [72]. The Pt/CrN electrocatalyst exhibited higher corrosion resistance and electrochemical active surface area (ECSA) than those of Pt/C. The superior electrocatalytic activity is attributed to the high conductivity of CrN support as well as the synergistic effect between Pt and CrN support. In another report, Yue et al. comparatively studied the electrocatalytic activity and durability between Pt/TiN and Pt/C. This work found that the Pt/TiN electrocatalyst displayed a 22% decrease in the initial ECSA, which was observably superior to that of Pt/C (66%) after 1000 cycles [73]. At the same time, the E_1/2_ of Pt/TiN (0.85 V) was also higher than that of Pt/C (0.84 V). Such encouraging performances were attributed to the fact that the energy levels between Pt-d and N-p states were similar, which facilitated the electron transfer to form Pt–N–Ti bonds.

In recent years, TMN has also been used as an ORR electrocatalyst. MoN/C and Mo_2_N/C electrocatalysts were synthesized and studied for the relationship between their structure and ORR performance [74]. This work revealed that the energy gap between the HOMO of MoN/C and the LUMO of O_2_ was excessively small, which brought a fast electron transfer between them. As a result, MoN/C exhibited higher ORR electrocatalytic activity compared with Mo_2_N/C in O_2_-saturated HClO_4_. In addition to the MoN-based electrocatalyst, a series of novel heterostructure Ni_3_N quantum dots (QD) were reported, which were uniformly distributed on the surface of NiO nanosheets [75]. According to the electronic structure characterizations, Ni_3_N QD showed a strong synergy effect with NiO nanosheets forming a unique electronic structure around the Fermi level, which not only increased the carrier concentration and conductivity, but also enriched the active sites of oxygen. Therefore, the ORR activity of the favorable Ni_3_N QD/NiO electrocatalyst was comparable to that of Pt/C.

#### 3.1.2. Multi-Metallic TMNs

Although the mono-metallic TMN displays impressive electrocatalytic activities, to further enhance the ORR performance, many scholars have conducted experiments on multi-metallic TMNs. Luo et al. explored the limitations of early-transition metal nitrides (ScN, TiN, CrN, and VN) as competitive electrocatalysts [76]. This work proposed a strategy to enhance the ORR activity by doping with transition metals and synthesized V_0_._95_M_0_._05_Ns (M = Cu, Ni, Co, Fe, Mn, Cr, and Ti). Among them, V_0_._95_Co_0_._05_N exhibited the most attractive ORR performance with the quasi-four-electron transfer, and the results were competitive with those of 20 wt.% Pt/C in an alkaline medium.

**Figure 4:**
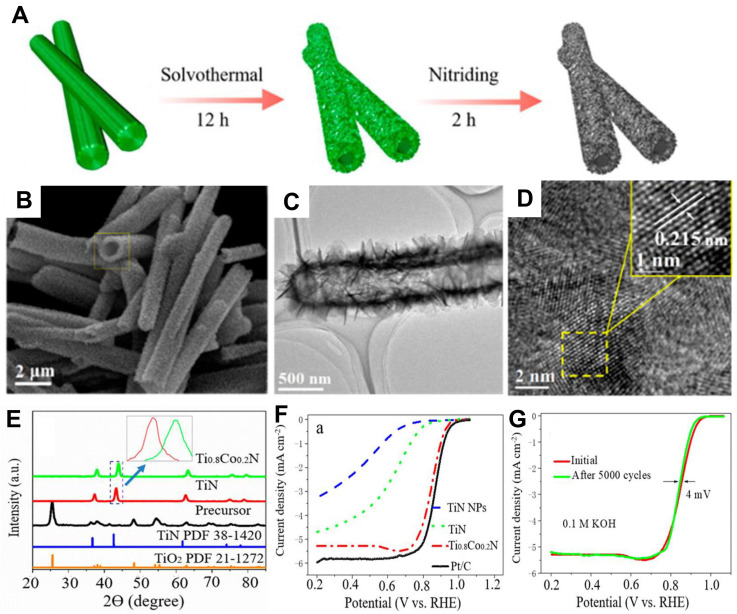
(**A**) Schematic of the formation of titanium nitrides assemblies. (**B**) SEM image of Ti_0_._8_Co_0_._2_N assemblies. (**C**–**D**) TEM images of Ti_0_._8_Co_0_._2_N assemblies. (**E**) XRD pattern of TiO_2_, TiN and Ti_0_._8_Co_0_._2_N assemblies. (**F**) LSV curves of different electrocatalysts in 0.1 M KOH electrolyte. (**G**) Potential cycling performance of Ti_0_._8_Co_0_._2_N assemblies. Reproduced with permission from [74]. Copyright © 2018 American Chemical Society.

Tian et al. investigated the electrocatalytic effects of the doping elements (Fe, Co, and Ni) on TiN [77]. The experimental data indicated that the doping might have an impact on the microstructure of the TiN with the decrease in the d-band vacancy of Ti atoms, which promotes the ability for providing electrons to oxygen. Most importantly, the ORR performance of Ti_0_._95_Ni_0_._05_N was almost comparable to that of commercial Pt/C in 0.1 M KOH. Later, a continuous study toward the effects of the doping cobalt on TiN for electrocatalytic performance was proposed [78]. The prepared Ti_0_._8_Co_0_._2_N nanosheets by the solvothermal with post-nitriding approach exhibited the electronic modification effect caused by Co doping with hollow nanostructure, endowing the Ti_0_._8_Co_0_._2_N electrocatalyst with a remarkable E_1/2_ of 0.85 V (Figure 4A–F). The resulting electrocatalyst revealed robust durability with only a 4 mV shifting of the E_1/2_ after the stability test for 5000 cycles (Figure 4G).

In contrast to Tian and co-workers, who prepared TMNs with Co doping on TiN as ORR electrocatalysts, Li et al. synthesized Ti_0_._95_Fe_0_._05_N support with a large specific surface area and high conductivity with a hydrothermal method followed by post-nitriding treatment [79]. As a result, Pt/Ti_0_._95_Fe_0_._05_N displayed higher activity and stability than that of Pt/C. In other words, it was obvious that the ECSA of Pt/C and Pt/Ti_0_._95_Fe_0_._05_N decreased by 29% and 7% after 1000 cycles, and it diminished by 90% and 30% after 5000 cycles, respectively. Furthermore, introducing Fe into TiN nanotubes enhanced the synergy effect between metal species, which resulted in the intensified activity of the Pt/Ti_0_._95_Fe_0_._05_N nanotube electrocatalyst.

In general, it can be seen from this section that TMNs are promising materials to improve ORR electrocatalysis performance, especially in an alkaline environment. However, the relatively poor activity and durability of TMNs in acidic medium is an issue that urgently needs to be solved.

### 3.2. Transition Metal Nitrides as OER Electrocatalysts

There is no doubt that noble metal (Ir and Ru)-based materials present satisfactory activity towards the OER process. However, the relative scarcity and high cost of noble metal-based electrocatalysts and the poor stability of Ru-based electrocatalysts make their application in this field unsustainable. Therefore, it is momentous to explore novel OER electrocatalytic materials with encouraging lower overpotential, superior stability, and lower cost. In recent years, TMNs have attracted a great deal of attention due to their electronic structure, which is similar to that of precious metals [80]. Detailed OER performances are summarized in Table 1.

#### 3.2.1. Mono-Metallic TMNs

The Co-based nitride electrocatalyst exhibits strong electron-donating ability and high durability derived from the change of the M–N bond and the state density of the metal d-band. Co_4_N porous nanowires based on carbon cloth were prepared, and the electrocatalyst revealed superior activity for OER with an overpotential at 10 mA cm^−2^ of 257 mV, which benefited from the synergy effect of metallic property, 1D porous nanowire arrays, and unique 3D electrode configuration [81]. Later, a novel method was proposed to prepare cobalt nitride nanowires through N_2_ radio-frequency plasma treatment [82]. In this way, it took only 1 min to obtain cobalt nitride.

In addition to cobalt-based TMN electrocatalysts, nickel-based and iron-based TMN electrocatalysts were also employed in the field of OER processes. Ni_3_N nanosheets of OER electrocatalysts were developed by Xu and co-workers [83]. The Ni_3_N nanosheets performed well in OER with an overpotential of 350 mV and Tafel slope of 45 mV dec^−1^ for abundant active sites as well as favorable conductivity. Surface plasmon resonance (SPR) was a recognized mechanism by which to enhance the activity of OER. Zeng et al. developed a cation exchange strategy to prepare Fe_2_N nanoparticles embedded in mesoporous TiO*_x_*N*_y_* nanoshells, which could be used as a plasmonic material to induce hot electrons and possess high porosity and electrical conductivity [84]. The Fe_2_N nanoparticles served as an efficient OER electrocatalyst in an alkaline medium with an extremely small overpotential of 270 mV at the current density of 10 mA cm^−2^. The robust TMNs also display great potential as support for noble metal nanoparticles. Li et al. employed TiN as support for effectively loading IrO_2_@Ir nanoparticles. TMN support also modifies the electronic feature of iridium by downshifting its d-band center, which promotes both OER activity and stability.

#### 3.2.2. Multi-Metallic TMNs

To improve the OER performance of TMNs, many scholars tend to introduce heteroatoms into mono-metallic TMNs to change the electronic structure, optimize the coordination of the active metal center environment, and improve the intermediate adsorption as well as desorption on the interface.

**Figure 5:**
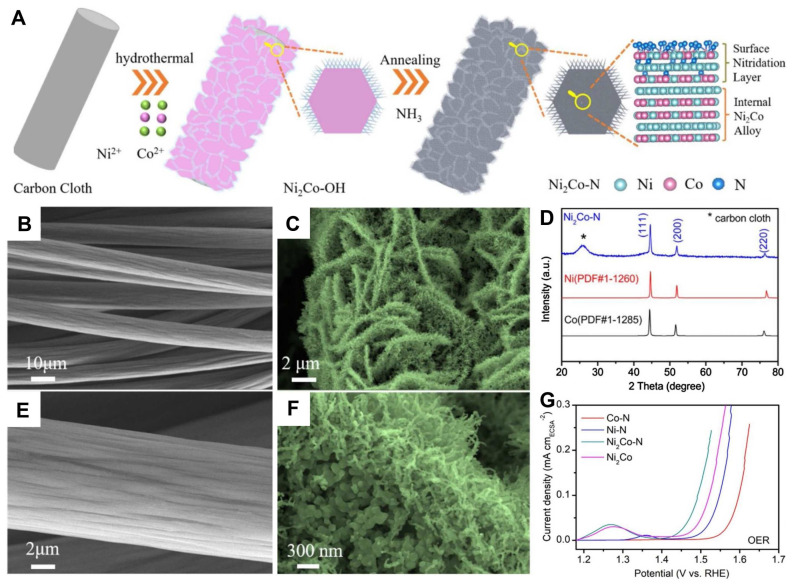
(**A**) Schematic illustration of the formation of Ni_2_Co-N nanocactoids grown on carbon cloth; SEM images of (**B**,**C**) bare carbon cloth; (**D**,**E**) Ni_2_Co-N; (**F**) XRD pattern of Ni_2_Co-N; (**G**) LSV curves of different electrocatalysts in 0.1 M KOH electrolyte. Reproduced with permission from [82]. Copyright © 2020 Elsevier.

**Figure 6:**
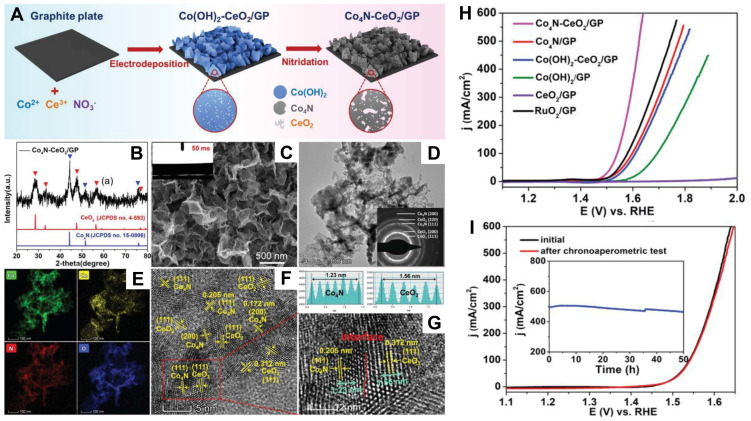
(**A**) Schematic of the formation of the Co_4_N-CeO_2_/GP; (**B**) XRD pattern of Co_4_N-CeO_2_/GP; (**C**–**G**) HRTEM image of CeO_2_, Co_4_N, and Co_4_N-CeO_2_/GP; (**H**) LSV curves of different electrocatalysts in 0.1 M KOH electrolyte; (**I**) Potential cycling performance of Co_4_N-CeO_2_/GP. Reproduced with permission from [85]. Copyright © 2020 Wiley-VCH Verlag.

Wang and co-workers grew nickel–cobalt nitride nanosheets on macroporous Ni foam (NF) by electrodeposition [85]. The obtained NiCo_2_N-NF electrocatalyst achieved robust stability and promising activity for OER with an overpotential of 290 mV at 10 mA cm^−2^ due to its 3D, interconnected porous structure and the synergistic effect of bimetallic active sites. A surface nitridation strategy was studied with the synergistic effect of nickel–cobalt [86]. The synthesized nickel–cobalt nitride (Ni_2_Co-N) nanocactoids on carbon cloth exhibited a remarkable activity with an overpotential of 214 mV and a Tafel slope of 53 mV dec^−1^ in alkaline media, much lower than the results of commercial IrO_2_ (Figure 5). Li et al. offered a facile approach to acquire holey cobalt-iron nitride nanosheets based on Ni foam substrate [87]. The obtained CoFeN*_x_*HNAs/NF served as an attractive OER electrocatalyst with a large ECSA, low charge transfer resistance, and rapid mass diffusion. In addition, the electrocatalyst showed a low overpotential of 260 mV. Moreover, the effect of the molar ratio was investigated in the iron atoms and cobalt atoms on the Fe-CoN electrocatalyst [88]. The results showed that the optimal CoFe(3:1)-N had remarkable activity for OER, with an overpotential of 200 mV and excellent durability. As shown in Figure 6A–C, Sun et al. took advantage of the synergistic effect of Co_4_N/CeO_2_ to prepare a Co_4_N-CeO_2_ hybrid nanosheet array grown on a graphite plate (Co_4_N-CeO_2_/GP) by anion intercalation enhanced electrodeposition route and subsequent nitridation [89]. The treatment of CeO_2_ coupling Co_4_N porous nanosheet significantly enhanced the OER activity, which was evidenced by a low overpotential of 239 mV to reach a current density of 10 mA cm^−2^ and long-term durability at a large current density of 500 mA cm^−2^ for 50 h (Figure 6D,E).

**Figure 7:**
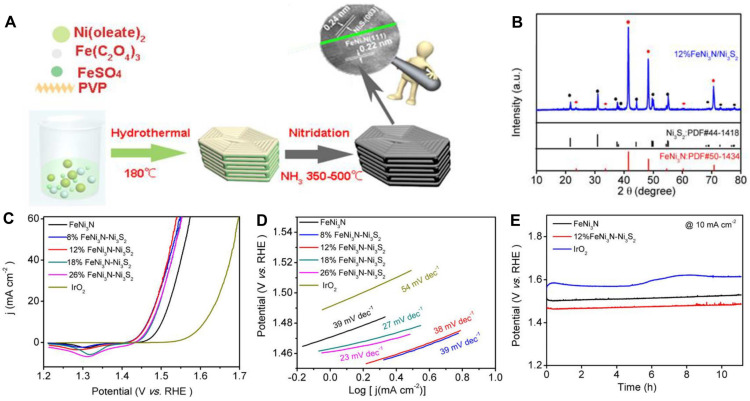
(**A**) Schematic illustration of the formation of FeNi_3_N-Ni_3_S_2_ samples; (**B**) XRD pattern of FeNi_3_N-Ni_3_S_2_; (**C**) LSV curves of different electrocatalysts in 0.1 M KOH electrolyte; (**D**) Tafel plots of different electrocatalysts; (**E**) potential cycling performance of FeNi_3_N-Ni_3_S_2_, FeNi_3_N, and IrO_2_. Reproduced with permission from [91]. Copyright © 2020 American Chemical Society.

For nickel-iron bimetallic TMN electrocatalysts, two-dimensional and nanocrystalline Fe_2_Ni_2_N/rGO nanohybrid sheets were developed [90]. The resulting electrocatalyst shows superior activity with an overpotential of 290 mV at 10 mA cm^−2^ and robust long-term stability for over 24 h, the encouraging OER performance was triggered by the synergistic effect between nanocrystalline Fe_2_Ni_2_N and graphene nanosheets as well as its unique nanoarchitecture. Moreover, FeNi_3_N-Ni_3_S_2_ electrocatalyst was obtained by hydrothermal and nitridation processes (Figure 7A,B) [91], and the obtained electrocatalyst reveals brilliant OER activity with an overpotential of 230 mV and a Tafel slope of 38 mV dec^−1^. The electrocatalyst showed no obvious decay over 40,000 s (Figure 7C–E). The high ECSA and the electron effect between FeNi_3_N and Ni_3_S_2_ were also studied, which reduced the activation energy of the OER process and thus enhanced the intrinsic activity of the electrocatalyst. To further prove that the Fe doping modified the electronic structure of Ni_3_N and improved the redox activity of the OER electrocatalyst surface, Ni_3_FeN was grown on a 3D network-like support of the strutted graphene foam (Ni_3_FeN/SG) [92]. The electrocatalyst shows enhanced OER activity, as evidenced by a low overpotential of 226 mV at 10 mA cm^−2^. It should be noted that the 3D structure increased the ECSA and promoted active site exposure, which helped the diffusion of the reactants and accelerated the electrocatalytic OER process. A similar approach can be compounded with a NiFeOOH/Ni_3_FeN/Ni heterojunction and the importance of heterojunction in improving OER activity was stressed [93]. The obtained electrocatalyst showed attractive performance for OER, which was largely beneficial for charge transfer. Such electrocatalyst only required an overpotential of 200 mV to actuate 10 mA cm^−2^, superior to most recently reported electrocatalysts and commercial RuO_2_.

Furthermore, Ni-doped molybdenum nitride nanorods were synthesized as OER electrocatalysts, which not only performed a remarkable overpotential of 218 mV but also maintained long-term stability for over 110 h [94]. It also indicated that TMNs in combination with multiple active components could form a heterostructure that enhances electrocatalytic performance.

### 3.3. Transition Metal Nitrides as Bifunctional ORR&OER Electrocatalysts

Until now, Pt-based electrocatalysts have been considered the best ORR electrocatalysts. However, Ir/Ru-based electrocatalysts have been the best choices for OER. However, pure Pt-based or Ir/Ru-based materials are too expensive and not sufficiently active for bifunctional OER and ORR. Hence, TMN-based materials serving as highly active and stable bifunctional electrocatalysts have been widely investigated in recent years [95,96]. In addition, the ORR&OER properties of recently reported bifunctional electrocatalysts based on TMNs are shown in Table 2.

A novel bifunctional electrocatalyst (Ni_3_FeN/NRGO) was prepared [97], which was promising for the replacement of commercial noble-metal electrocatalysts. The NRGO not only dispersed the Ni_3_FeN nanoplates, but also provided a conductive framework for the high retention of the ECSA. By coupling the theoretical and experimental approaches, Ni_3_FeN/NRGO was confirmed to show excellent performance in both OER and ORR with the lowest onset overpotential of 150 mV for OER and the highest onset potential of 0.9 V for ORR among all samples. In addition, a novel bifunctional Ni_3_FeN/Co,N-CNF electrocatalyst was delivered driving both ORR and OER [98]. Because of Co,N-CNF as the support of the electrocatalyst, the Ni_3_FeN nanoparticles were highly dispersed. As a result of the synergistic effect of Ni_3_FeN and Co,N-CNF, the prepared electrocatalyst presented better OER and ORR activities than those of noble metal electrocatalysts with the low overpotential of 270 mV for OER and E_1/2_ of 0.81 V for ORR. Zhang et al. also reported ultra-small nanoparticles (Fe_2_N) grown on CNTs. Compared with the pure Fe_2_N nanoparticles, the Fe_2_N nanoparticles in Fe_2_N-CNTs were much smaller (5 nm) [99]. Thus, the E_1/2_ of Fe_2_N-CNTs for ORR was 0.71 V and the overpotential for OER was 240 mV. This work might bring us a novel and environmentally friendly approach to the preparation of ultra-small TMNs.

**Figure 8:**
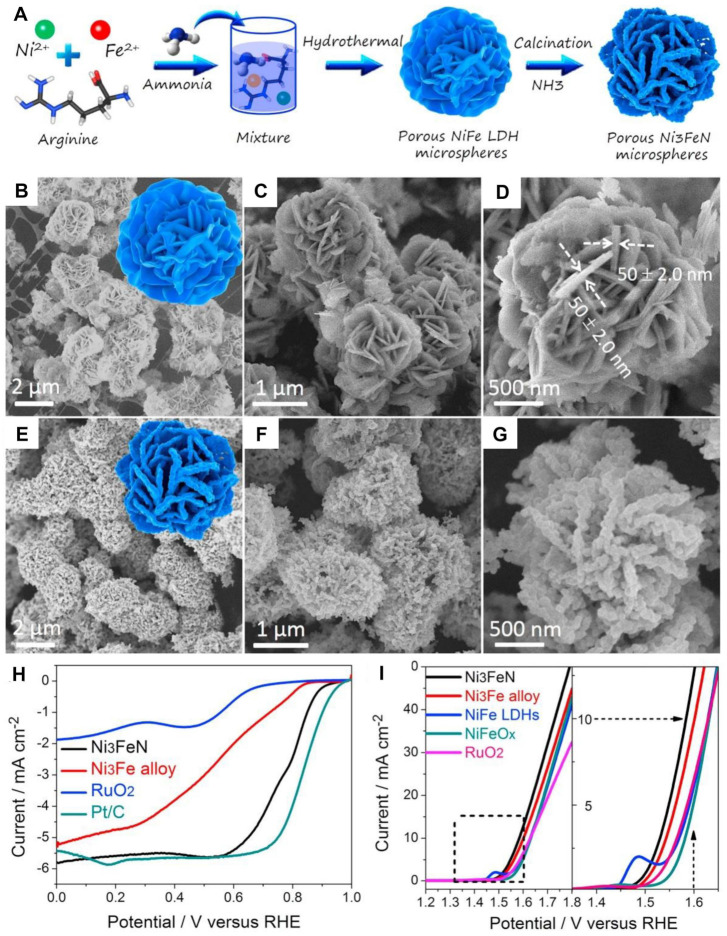
(**A**) Schematic of the formation of the porous Ni_3_FeN hierarchical microspheres; (**B**–**D**) SEM images of the NiFe LDH hierarchical microspheres; (**E**–**G**) SEM images of the porous Ni_3_FeN hierarchical microspheres; (**H**) LSV curves in 0.1 M KOH electrolyte for ORR; (**I**) LSV curves in 0.1 M KOH electrolyte for OER. Reproduced with permission from [100]. Copyright © 2017 Elsevier.

To further improve the stability of nickel–iron nitride electrocatalyst, a mesoporous nickel–iron nitride bifunctional electrocatalyst was prepared without carbon support (Figure 8A) [100]. The Ni_3_FeN electrocatalyst was microspheric with a hierarchically porous structure, which provided abundant interparticle void space and a high specific surface area (Figure 8B–G). In this manner, the resulting sample showed excellent ORR activity with an E_1/2_ of 0.78 V and OER activity with a low overpotential of 355 mV at 10 mA cm^−2^ in an alkaline medium (Figure 8H,I). A trimetallic (NiFeMn) nitride electrocatalyst was produced in a molecular sheet form, which was stabilized by Ti metal on titanium carbide (Ti_3_C_2_) sheets [101]. Intimate contact between the two sheets produced a strong force at the interface, thus effectively avoiding the accumulation of a nitride sheet. The resulting bifunctional electrocatalyst exhibited the lowest ΔE (ΔE = E_j = 10_ − E_1/2_) of 0.68 V and stable discharge–charge cycling over 120 h. This work unlocked a high electrocatalytic performance of trimetallic nitride electrocatalyst and provided a new way for the application of 2D sheets in flexible and wearable devices. In contrast to the above, Fe_3_Pt electrocatalysts with porous nickel–iron nitride as support were prepared [102]. On the one hand, unlike the widely studied Pt-M disordered alloys, ordered Fe_3_Pt intermetallic alloy possessed definite composition and structure, which was beneficial for the high dispersion of active sites. On the other hand, Ni_3_FeN support showed brilliant chemical stability and high conductivity. Therefore, the Fe_3_Pt/Ni_3_FeN electrocatalyst released excellent electrocatalytic activity for both OER and ORR and achieved a long-term cycling performance over 480 h at 10 mA cm^−2^ in Zn-air batteries.

**Figure 9:**
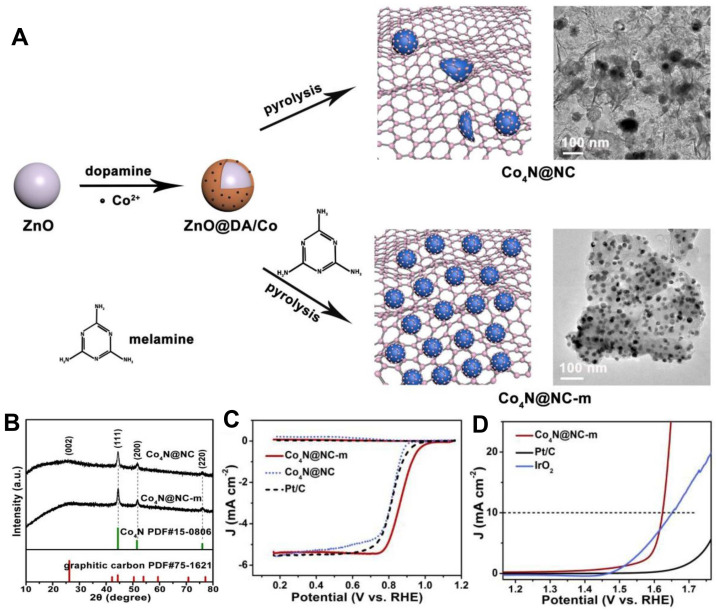
(**A**) Schematic of the formation of Co_4_N@NC-m and Co4N@NC; (**B**) XRD patterns of Co_4_N@NC-m and Co_4_N@NC; (**C**) LSV curves in 0.1 M KOH electrolyte for ORR; (**D**) LSV curves in 0.1 M KOH electrolyte for OER. Reproduced with permission from [100]. Copyright © 2019 Elsevier.

Chen and co-workers obtained Co_4_N@N-doped carbon (Co_4_N@NC-m) with the assistance of melamine [103]. Moreover, melamine not only acted as a nitrogen doping agent, but also helped increase conductivity and ECSA of the electrocatalyst and regulated the size and distribution of Co_4_N nanocrystals (Figure 9). Therefore, Co_4_N@NC-m served as a high-activity and long-term stability electrocatalyst for both ORR/OER and air cathode of rechargeable Zn-air batteries. In addition, this work provided an effective approach to synthesizing non-noble metal electrocatalysts with controllable morphology. Guan et al. developed cobalt nitride nanoparticles supported on a nitrogen-doped reduced graphene oxide sheet (O-S-Co_5_._47_N@N-RGO) [104]. Such flexible material showed satisfactory electrocatalytic activity toward both OER and ORR with low overpotentials 380 mV at 10 mA cm^−2^ current density and E_1/2_ of 0.82 V in 1 M KOH solution. It was believed that the Co-N sites in the RGO sheet and the Co sites on the surface of O–S–Co_5_._47_N crystal were the active sites for ORR and OER, respectively. Cobalt-iron bimetallic nitrides with N-doped multi-walled carbon nanotubes (Co-Fe-N@MWCNT) were developed [105]. It is worth noting that MWCNTs acted as a bridge to connect nanoparticles to optimize the conductivity of the electrocatalyst, while regulating the uniform dispersion of nanoparticles to increase ECSA. In addition, there was a strong interaction between iron and cobalt, and iron could effectively regulate the conversion of cobalt from cobalt (III) to cobalt (IV). Thus, Co-Fe-N@MWCNT performed well for both OER and ORR.

### 3.4. Design Principles of TMNs Electrocatalysts for Oriented Applications

TMNs play a key role in electrocatalytic application, especially in OER and ORR. The oxygen-involved reactions require extremely abundant accessible electrocatalytic sites due to their slow kinetics. Thus, the reasonable design of TMNs is an effective approach to improving the performance of the electrocatalytic application.

In the aspect of doping agents, the electrocatalyst is desired to adjust the electronic structure to improve the ability to donate electrons to adsorbed oxygen. Furthermore, the doping agents show great influence on the microstructures of electrocatalysts. With the defects produced, active centers are formed, which is beneficial to capture electrons and speed up electron transport.

On the other hand, the electrocatalyst is expected to possess a large number of exposed active sites because the electrocatalytic reaction takes place on the electrochemical surface of the electrocatalyst. Hence, suitable nanostructures with a high specific surface area, such as nanoflowers and hierarchical porosity, is required to take full advantage of exposed active sites. A strong synergy effect between the intrinsic activity and nanostructures is also demanded to achieve the improvement of electrocatalytic performance.

## 4. Conclusions and Perspectives

In this review, we summarized the recent advances in the synthesis and application of transition metal nitrides for oxygen reduction reaction and oxygen evolution reaction. Selecting different transition metal ions (Ti, Ni, Fe, Co, Mo) or introducing heteroatoms into the unary transition metal could make the TMNs produce more similar properties to noble metals. Moreover, a high-efficiency multiscale mass transfer structure was constructed by using TMN nanowires and porous structures, which has been proven to greatly enhance the electrocatalytic performance of TMNs. To develop the preparation and application of TMNs, the following challenges need to be studied later.

First, the preparation of TMN electrocatalysts has been restricted to laboratory preparation, which cannot be produced on a large scale due to its complicated preparation and high cost. Therefore, simple, low-cost, and environmentally friendly preparation methods should be investigated as a part of the subsequent research.

Secondly, in acidic media or under an environment with extreme pH value conditions, the activity of TMN electrocatalysts will be severely reduced due to the dissolution of transition metals, protonation of active sites, and corrosion of conductive substrates. Therefore, TMNs electrocatalysts with robust stability need to be developed.

Thirdly, to further improve the electrocatalytic activity, the electronic structure can be adjusted based on the d-band center theory by doping with metal or non-metal elements, forming a heterostructure, or based on the synergistic effect. Moreover, the density functional theory calculation for multi-metallic TMNs is rare, and the systematic calculation to investigate the electrocatalytic mechanism is of great significance to optimize active sites and their evolution process.

TMNs have been studied in all dimensions, but there is a lack of comparison between TMNs in various dimensions. Numerous works can be carried out in this field to investigate the most suitable dimension under various electrocatalytic conditions. Moreover, combining multiple dimensions may result in exceptional performance.

Finally, in-situ techniques like XRD/ND-analysis, TEM, and XPS are used to monitor the changes of TMNs during the preparation process and the electrocatalytic reaction process, which is beneficial for understanding the principle of TMN catalysis and provides a theoretical basis for subsequent electrocatalyst researches.

## Figures and Tables

**Table 1:** OER electrocatalytic performance of recent advanced TMNs electrocatalysts.

Catalysts	Morphology	Electrolyte	E_j=10_ [V vs. RHE]	Tafet Slope [mV dec^−1^]	Ref.
Co_4_N	1D	1 M KOH	1.49	44	[81]
CoN	1D	1 M KOH	1.52	70	[82]
Ni_3_N	2D	1 M KOH	1.58	45	[83]
TiO_x_N_y_-Fe_2_N	3D	1 M KOH	1.54	59	[84]
NiCo_2_N/NF	1D	1 M KOH	1.52	65	[85]
Ni_2_Co-N	3D	1 M KOH	1.44	53	[86]
CoFeN*_x_*HNAs /NF	2D	1 M KOH	1.49	57	[87]
CoFe_(3:1)_-N	3D	1 M KOH	1.43	42	[88]
Co_4_N-CeO_2_/GP	2D	1 M KOH	1.47	46	[89]
Fe_2_Ni_2_N/rGO	2D	1 M KOH	1.52	49	[90]
FeNi_3_N-Ni_3_S_2_	3D	1 M KOH	1.46	38	[91]
Ni_3_FeN/SG	2D	1 M KOH	1.46	43	[92]
NiFeOOH /Ni_3_FeN/Ni	3D	1 M KOH	1.43	36	[93]
Nifoam@Ni- Ni_0_._2_Mo_0_._8_N	1D	1 M KOH	1.45	39	[94]

**Table 2:** Summary of the OER&ORR activities of recently reported electrocatalysts based on TMNs (the voltage gap: ΔE = E_j__= 10_ − E_1/2_).

Catalysts	Morphology	Electrolyte	E_1/2_ for ORR [V]	E_i = 10_ for OER [V]	ΔE [V]	Ref.
Ni_3_FeN/NRGO	2D	0.1 M KOH	0.72	1.38	0.77	[97]
Ni_3_FeN/Co,N-CNF	0D	0.1 M KOH	0.81	1.50	0.69	[98]
Fe_2_N/N-CNTs	0D	0.1 M KOH	0.71	1.66	0.95	[99]
Ni_3_FeN	3D	0.1 M KOH	0.78	1.58	0.70	[100]
NiFeMnN	2D	0.1 M KOH	0.84	1.52	0.68	[101]
Fe_3_Pt/Ni_3_FeN	3D	0.1 M KOH	0.93	1.60	0.72	[102]
Co_4_N@NC-m	0D	0.1 M KOH	0.87	1.63	0.81	[103]
Co_5_._47_N	0D	0.1 M KOH	0.82	1.61	0.80	[104]
Co-Fe-N@MWCNT	0D	0.1 M KOH	0.92	1.52	0.72	[105]

## Data Availability

All raw data in this study can be provided by the corresponding authors on request.
